# The Histomorphological Spectrum of Placenta in Growth Restricted Fetuses in A Tertiary Care Centre in South India

**DOI:** 10.30699/IJP.2023.551426.2867

**Published:** 2023-03-22

**Authors:** Abinaya Sundari Amirthakatesan, Sharmila Devi Chandramohan, Uma maheshwari Gurusamy

**Affiliations:** 1 *Department of Pathology, PSG IMSR, Coimbatore, India *; 2 *Department of Professor, Pathology, Annapoorana medical college, Salem, India*; 3 *Professor, Pathology, PSG IMSR, Coimbatore, India*

**Keywords:** Distal Villous Immaturity (DVI), Fetal Growth Restriction (FGR), Fetal Vascular Malperfusion (FVM), Maternal Vascular Malperfusion (MVM), Placental examination

## Abstract

**Background & Objective:**

Fetal growth restriction (FGR) is one of the leading causes of perinatal morbidity and mortality. Our study aimed to analyze the gross and histopathological changes in the placentas of growth-restricted fetuses.

**Methods:**

Placentas of fifty growth-restricted fetuses received in the Department of Pathology for 3 years were studied. Clinical data including ultra-sonographic findings were obtained. The received placentas were photographed and the details were documented in a prepared template. The relevant tissues were processed, analyzed, and correlated with the clinical findings.

**Results:**

The study demonstrates distinctive gross and histological abnormalities in the placentas of growth-restricted fetuses. More than two-thirds of the placentas had shorter gestational age (preterm), seen as commonly associated with maternal co-morbidities such as oligohydramnios and pregnancy induced hypertension (PIH). The predominant gross lesions observed were the umbilical cord abnormalities, infarcts, and intervillous thrombus. Maternal vascular malperfusion (MVM) and fetal vascular malperfusion (FVM) were the two common histologic findings. Characteristic placental lesions with a significant risk of recurrence identified were distal villous immaturity (DVI), villitis of unknown etiology (VUE), and massive perivillous fibrin deposition (MPVFD). The unusual placental causes included villous capillary lesions and histological chorioamnionitis.

**Conclusion:**

Although a diverse etiology can cause FGR, the severity depends on the cumulative effects of multiple placental lesions. Hence, a meticulous placental examination is crucial for the effective management of growth-restricted fetuses in the current and subsequent pregnancies.

## Introduction

The growth of the fetus is the outcome of the complex processes requiring unified functions of the maternal, fetal, and placental components (1). The placenta is the mediating organ between maternal and fetal circulations. Any imbalance in this system may result in fetal growth restriction. Fetal growth restriction (FGR) is one of the leading causes of perinatal morbidity and mortality with an incidence of 5-10% of all pregnancies (2). 

Fetal growth restriction is defined as a failure of the fetus to gain appropriate weight for a given gestational age (3). FGR may be produced as a consequence of maternal, fetal, and placental factors or can be idiopathic (4,5,6). Growth restriction in fetuses can produce various consequences like perinatal asphyxia, polycythemia, chronic lung disease, and chronic necrotizing enterocolitis (7) in neonatal life. Whereas in adult life it causes obesity, hypertension, and diabetes mellitus (8).

Integrated prenatal screening tests for serum markers and doppler study of uterine and umbilical arteries are emerging technologies for the identification of FGR (9). Regardless of the advanced technologies, diagnosing FGR is difficult owing to high false positive results. Therefore, placental pathology becomes valuable in early detection and management. Recent progress in placental specialty with standardized classification systems provides details about various placental pathologies in FGR (10).

In this study, we meticulously evaluated the gross and histologic features of the placentas of FGR fetuses. These features can improve our understanding of the pathophysiology of FGR and in addition explain the growth restriction of a fetus in the current pregnancy. It may also guide us to prevent the risk of recurrence in subsequent pregnancies and speculate on the future regarding targeted intervention.

## Material and Methods

The placentas of growth-restricted fetuses received in the Department of Pathology, PSG IMS & R during the study period of 3 years were taken. Ethics approval was obtained from the Institutional Human Ethics Committee. 

**Study Population**

The placentas of growth-restricted fetuses (Neonates with birth weight less than the 10th percentile) received were studied (11). Placentas of growth-restricted fetuses with congenital anomalies were excluded.

**Data Collection**

**Clinical Features**

A clinical proforma containing details like maternal age, gravidity, and parity, gestational age, obstetric history, maternal co-morbidities (Pregnancy induced hypertension(PIH), Gestational diabetes mellitus (GDM), oligohydramnios, thyroid diseases, autoimmunity, premature rupture of membranes, cardiac/ anemia complicating pregnancy), ultrasound details (Doppler study of the umbilical artery), details about the present and previous pregnancies, mode of delivery, neonatal details such as birth weight and neonatal intensive care admission was issued to the Department of Obstetrics and Gynaecology wherein, they fill the clinical details. The other clinical details were collected through the hospital information system.

**Gross Examination**

The specimens were sent either fresh or fixed in neutral buffered formalin. Photographs of the cord, fetal surface, maternal surface, and cross-sections of parenchyma with and without specific lesions were taken. A template was prepared for the gross examination. The template included features under the following headings.

1. Umbilical cord - color, length, insertion, number of twists, number of vessels, presence of edema, false or true knots
2. Membranes – color, insertion, and rupture site
3. Fetal surface- vessels, nodularity, subchorionic hematoma
4. Maternal surface- succenturiate lobe/ yellow plaques/calcification
5. Placental parenchyma – spongy, firm, color, infarct/intervillous thrombus, retro-placental hematoma, any other lesions
6. Any other specific lesion

Trimmed placental weights were recorded (after trimming the attached membranes and a major portion of the umbilical cord leaving behind a small stump) and were compared with the standard weights for trimmed singleton placentas. Serial sectioning was done and the entire parenchyma was inspected for the presence of any lesions.

Two bits of membrane rolls with two bits of the umbilical cord (one bit from each end), relevant sections of the lesions, and two to three bits of full-thickness normal parenchyma (1 cm and 2 cm on either side of cord insertion) were taken.

**Histopathological Examination**

The tissues were processed and stained using hematoxylin and eosin stains. Special stains such as Perl’s, Von Kossa, Reticulin, and Verhoeff-Van Gieson stains were done when required. Then the slides were analyzed and categorized by trained pathologists based on the definitions and criteria suggested by the Amsterdam Placental Workshop group consensus (12).

The findings were broadly categorized as Fetal Vascular Malperfusion (FVM), Maternal Vascular Malperfusion (MVM), Distal Villous Immaturity (DVI), Villitis of Unknown Etiology (VUE), Massive Perivillous Fibrin Deposition (MPVFD) & Acute Chorioamnionitis. 

The microscopic findings under the FVM category included thrombosis of the Fetal vasculature, segmental avascular villi, villous stromal vascular karyorrhexis, stem vessel obliteration/fibromuscular sclerosis, and vascular ectasia. MVM findings were inclusive of infarct/ thrombohematoma, retroplacental hemorrhage, accelerated villous maturity/distal villous hypoplasia, and decidual arteriopathy. DVI is defined by a monotonous villous population (defined as at least 10 such villi) of villi with centrally placed capillaries and decreased vasculosyncytial membranes observed in placentas of more than 34 weeks of gestation. VUE is defined as the presence of lymphohistiocytic villitis without funisitis. Other categories were also classified based on the Amsterdam Placental Workshop group consensus (12)**.**

## Results

A total of 50 placentas of growth-restricted fetuses were received during the study period. In the study group, 98% of cases showed placental findings, 78% showed maternal co- morbidities and 2% showed fetal cause (twin pregnancy).

**FGR: Clinical Features**

The mean age of the mothers of growth-restricted fetuses was 27 years. About 64% of cases were delivered as preterm (<37 weeks) and 58% of cases were primigravida. Absent or reduced diastolic flow in the umbilical artery Doppler study was found in 13 cases (26%).

The predominant maternal co-morbidity associated with Fetal growth restriction was oligohydramnios, which was seen in 17 (34%) cases. Clinical characteristics of FGR placentas are summarized in Table 1.

Table 2 shows the mean placental weight, Fetal weight, and placenta-fetal weight ratio of all cases. 

**Table 1:** Clinical characteristics of FGR placentas

**Total numberofcases**=50			
**Clinical details**		**No.of** **cases**	**Frequency** (%)
Mean age(years)		27	
Gestational age at delivery	<37weeks	35	70
	>37weeks	15	30
Obstetric history (gravidity)	Primi	29	58
	Multi	21	42
USG–Reduced/absent diastolic flow		13	26
Maternalco-morbidities	Oligohydramnios	17	34
	PIH	14	28
	GDM	4	8
	Auto-immune Disorders (antiphospholipid antibody syndrome (APLA), systemic lupus erythematosus(SLE), rheumatoid arthritis, immune thrombocytopenic purpura (ITP).)	6	12
	AbruptioPlacentae	2	4
	Cardiac/anemia/uterine abnormalities complicate pregnancy	7	14
Combined causes (oligohydramnios with PIH, PIH with autoimmune disorders, PIH with GDM, abruption with PIH)		16	32

**Table 2:** Mean placental weight, fetal weight, and the placento-fetal weight ratio of all FGR cases

Total numberofcases=50		
Placental weight	SGA	46cases
	AGA	4cases
Mean placental weight		246gm
Mean fetal weight		1780gm
Meanplacentalco-efficient(placental weight: fetal weight)		0.14

**FGR: Placental Findings**

Of the study group, the most common placental changes identified were Maternal Vascular Malperfusion (MVM) in 32 cases (64%) followed by umbilical cord abnormalities found in 26 cases (52%). and Fetal Vascular Malperfusion (FVM) in 10 cases (20%). The placental histopathological changes seen in FGR are shown in Table 3.

**Umbilical Cord Abnormalities (n=26)**

**Fig. 1:**
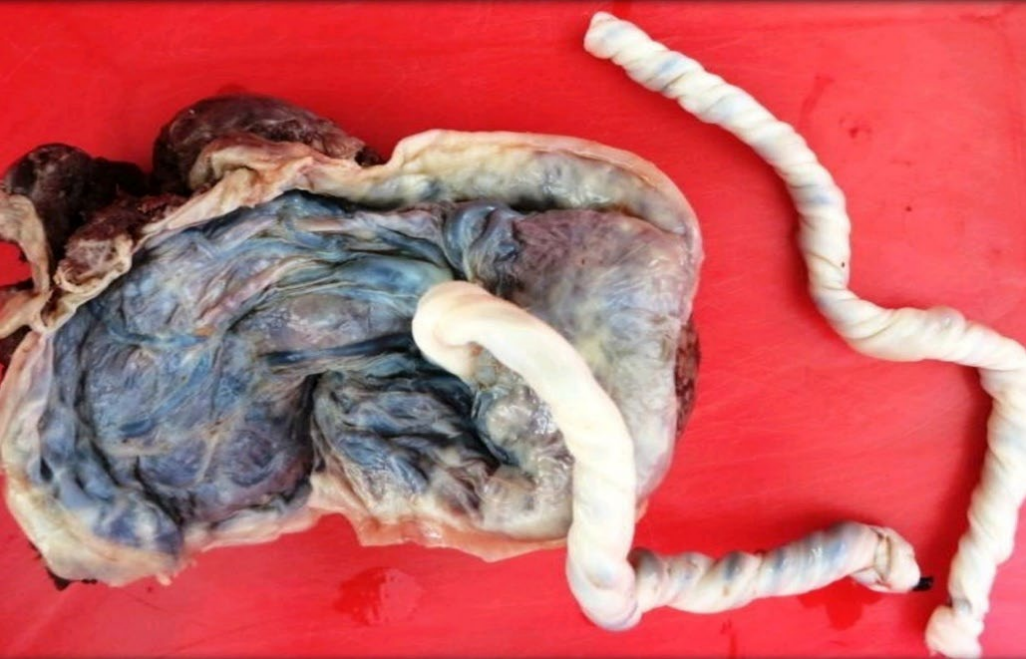
Umbilical cord abnormalities- Hypercoiled cord

**Fig. 2:**
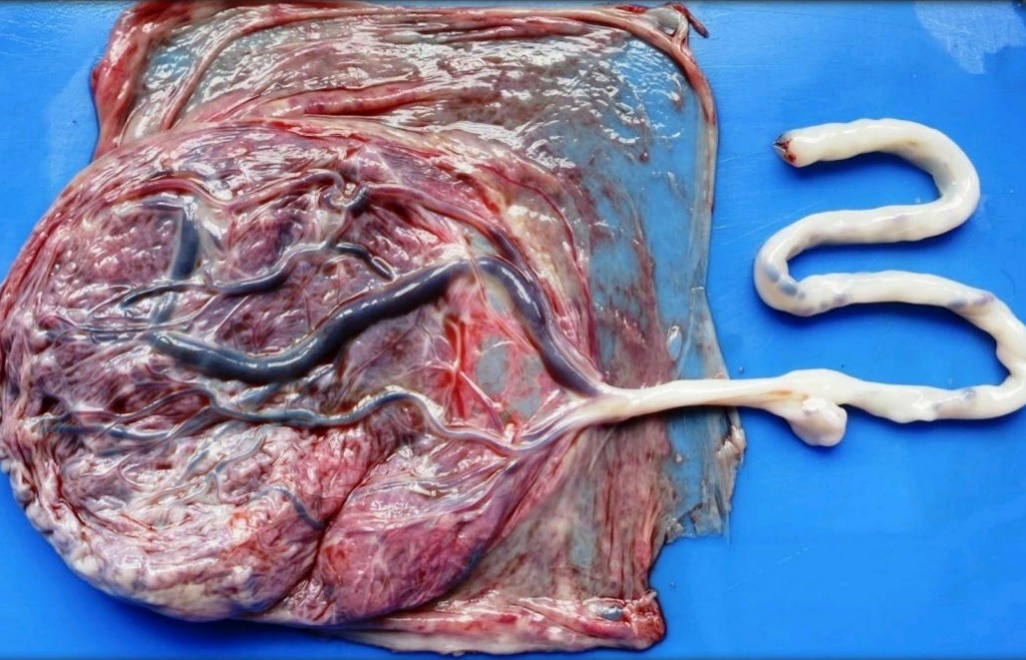
Umbilical cord abnormalities- Velamentous insertion of the cord

Table 4 shows the gross umbilical cord abnormalities found in our study group. We did not receive any case with a single umbilical artery, true knot, or excessively long cord in our study population(Figures 1 and 2).

**Table 3:** Placental histopathological changes seen in FGR

Placental causes(n=48)		
Placental-related causes	**No.of** **cases**	**Frequency**(%)
Cord abnormalities	26	52
Maternal vascular malperfusion	32	64
Fetal vascular malperfusion	10	20
FVM+MVM	7	14
FVM+umbilical cord abnormalities	5	10
Villous capillary lesions	9	18
Villitis of unknown etiology	8	16
Distal villous immaturity	6	12
Massive perivillous fibrin deposition	4	8
Acute chorioamnionitis	3	6
Abruption	2	4

**Table 4:** Gross umbilical cord abnormalities found in the study group

**Cord abnormalities **(n=26)			
**Lesions**		**No**. **of** **cases**	**Frequency**(%)
Coiling of cord	Hypercoiling	15	57.7
	Hypocoiling	8	30.8
Insertion of cord	Marginal	4	15.4
	Velamentous	5	19.2
	Paracentral/Eccentric	17	65.4
Placental weight	SGA	24	92.3
	AGA	2	7.7
Thin umbilical cord		1	3.8

**Maternal Vascular Malperfusion (MVM) (n=32)**

**Fig. 3:**
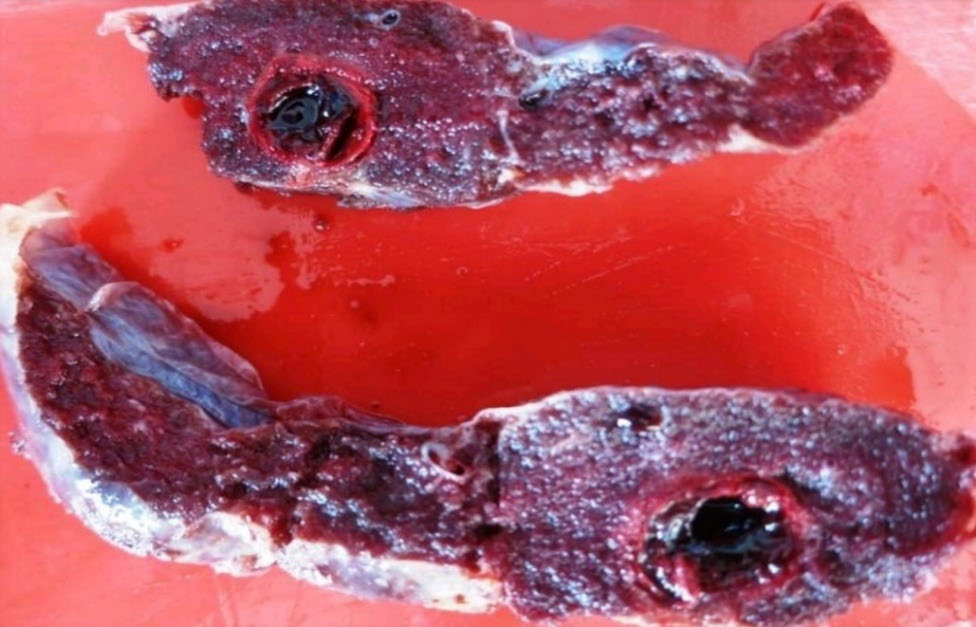
Maternal vascular malperfusion- Intervillous thrombus - Placental parenchyma shows a fairly circular blood clot that is red and shiny with a regular outline

**Fig. 4:**
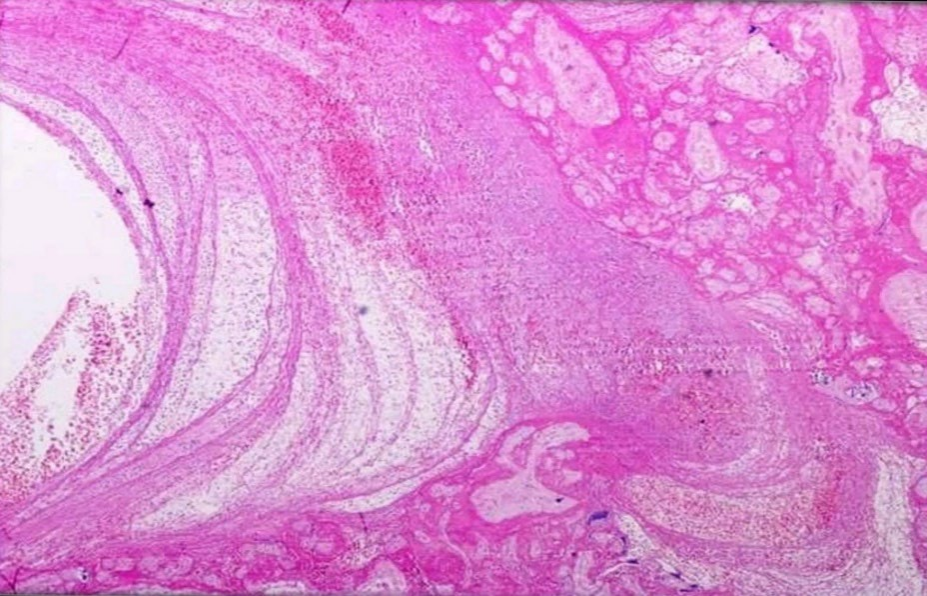
- Maternal vascular malperfusion- Low power view of a large intervillous thrombus shows laminated fibrin with blood elements and infarction in the adjacent villous tissue. (H&E, 4 X)

**Fig. 5:**
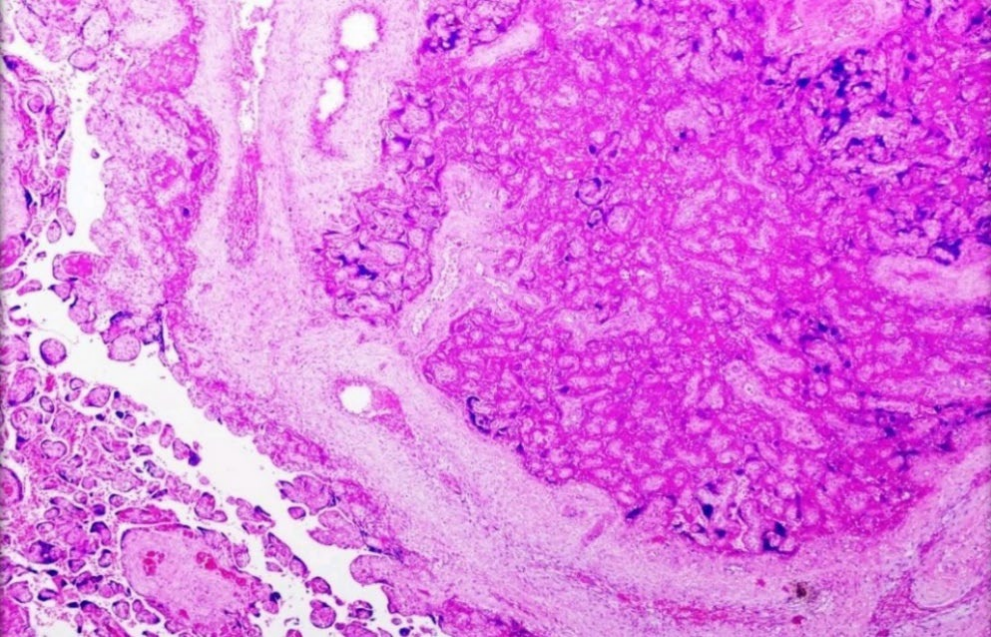
Maternal vascular malperfusion- A large focus of an infarct with collapsed villi, loss of intervillous space, and smudging of the trophoblastic nuclei. The viable villous tissue is seen at the left lower corner. (H&E, 4X)

**Fig. 6:**
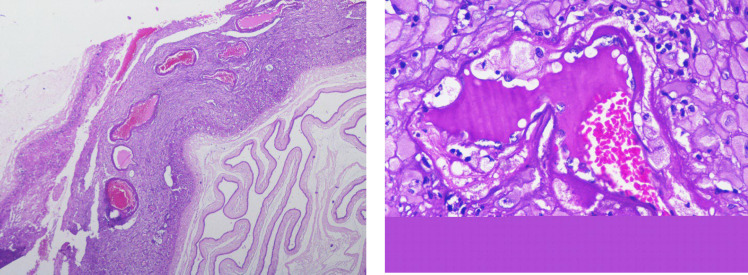
Maternal vascular malperfusion- Decidual vasculopathy: Membrane roll with characteristic thick-walled decidual vessels indicative of inadequate physiological remodeling of spiral arterioles. The arterioles show foamy macrophages with the tunica intima (atheroma) [H&E, (a): 4 X (b): 40 X]

Evidence of maternal vascular malperfusion was seen in 32 cases in which 18 cases were primigravida and 23 cases were preterm. The clinical correlates included 13 cases of PIH, 6 cases of oligohydramnios, 3 cases of GDM, and 5 cases of autoimmune diseases [Antiphospholipid Antibody Syndrome (APLA), Immune Thrombocytopenic Purpura (ITP) and Systemic Lupus Erythematosus (SLE)] 2 cases of abruption and 2 cases of cardiac diseases. 10 cases showed reduced/ absent diastolic flow in the umbilical artery doppler study. The gross and microscopic features of MVM in various cases are depicted in Table 5 (Figures 3, 4, 5, and 6).

**Table 5:** Gross and microscopic features of maternal vascular malperfusion

Maternal vascular malperfusion(n=32)				
	**Lesions**		**No.of cases**	**Frequency**(%)
Gross	Infarcts		17	53.1
	Intervillous thrombus		13	40.6
	Retroplacental hemorrhage		5	15.6
	Placental weight	SGA	29	90.6
		AGA	3	9.4
Microscopy	Increased syncytial knots		30	93.8
	Villous agglutination		14	43.8
	Peri villous fibrin deposition		20	62.5
	Distal villous hypoplasia		22	68.8
	Decidual vessels with acute atherosis/fibrinoid necrosis of the vessel wall		9	28.1
	Muscularized basal plate vessels		0	0
Increased cell islands		27	84.4

**Fetal Vascular Malperfusion (FVM) (n = 10)**

**Fig. 7:**
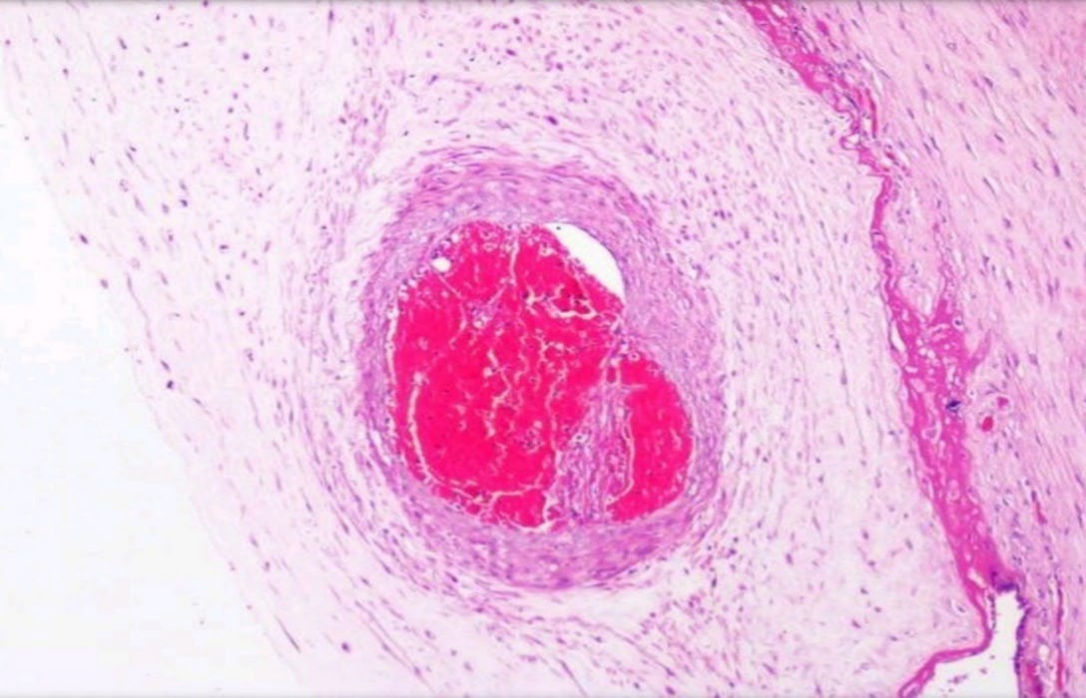
Foetal vascular malperfusion Chorionic plate vessel occluded by blood clot with clumps of fibrin. (H&E, 10 X)

**Fig. 8:**
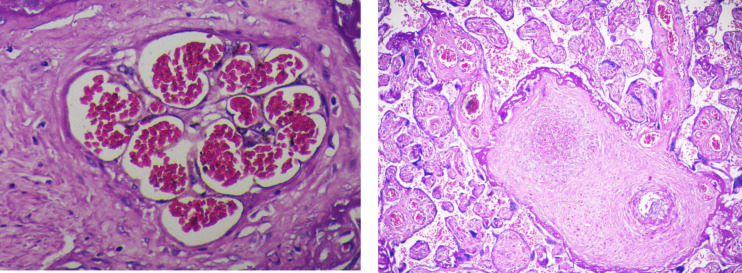
Foetal vascular malperfusion- a-High power view of the placental parenchyma with recanalizing thrombi in stem villi vessels. [H&E, 40 X] b- Hemorrhagic endo vasculitis - Stem villous vessel shows extravasated erythrocytes into the vessel wall with the presence of karyorrhectic debris. (H&E, 40 X)

**Fig. 9:**
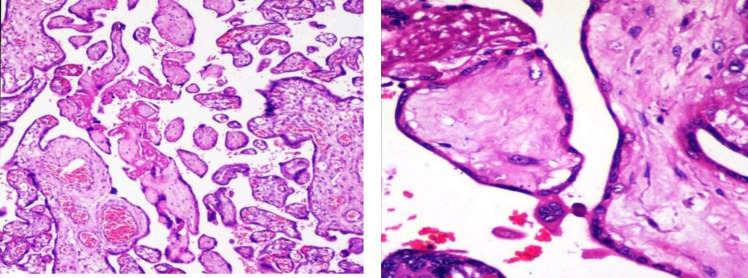
Foetal vascular malperfusion- A sharply demarcated focus of a group of avascular hyalinized villi with the absence of vessels in the villous stroma. (H&E) (a)10 X (b) 40X

Out of 50 cases, Fetal vascular malperfusion was present in 10 cases. All cases except one were delivered as preterm. Regarding clinical information, 2 cases presented with PIH, 1 case with GDM, and 2 cases with autoimmune diseases (ITP and SLE). In the Doppler study, 3 cases showed absent/ reduced diastolic flow in the umbilical artery. Table 6 enumerates major gross and microscopic features found in FVM (Figures 7, 8, and 9). 

**Table 6:** Gross and microscopic findings in fetal vascular malperfusion

**Fetalvascular malperfusion (n=10)**					
	**Lesions**			**No.of** **cases**	**Frequency** **(%)**
**Gross**	Thrombi in umbilical/chorionic vessels			**0**	**0**
	Placental weight		SGA	**10**	**100**
			AGA	**0**	**0**
**microscopy**	Vascular ectasia in chorionic plate/stemvilli			**6**	**60**
	Groups of avascular hyalinized villi			**9**	**90**
	Villous stromal karyorrhexis			**6**	**60**
	NucleatedRBCs			**1**	**10**
	Fibrin/re-canalizing thrombi	Umbilical vessels		**1**	**10**
		Chorionicplatevessels		**4**	**40**
Stemvillivessels		**8**	**80**

**Miscellaneous Placental Causes**

**Fig. 10:**
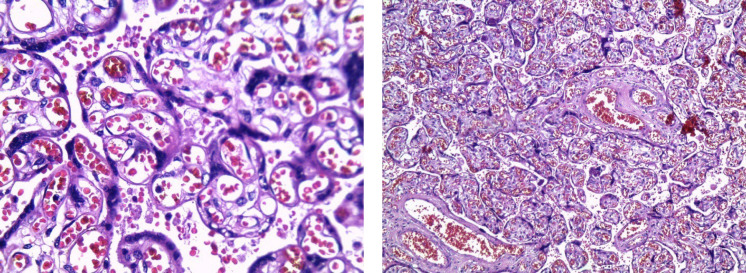
Villous capillary lesions. (H&E, 10X) (a) Chorangiosis – increased number of capillaries (more than 10/villi) in terminal villi (b) Chorangiomatosis – stem and intermediate villi show an increased number of vessels with prominent pericytes and stromal cells

**Fig. 11:**
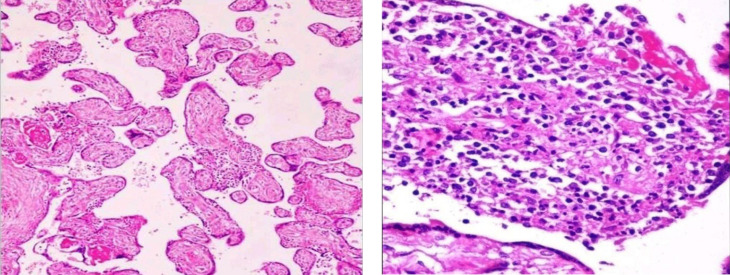
Villitis of unknown etiology (H&E) (a): Patchy, mild lymphohistiocytic infiltration of the villi (10 X) (b): The villi are expanded, invaded, and destroyed by chronic inflammatory cells. (40 X)

**Fig. 12:**
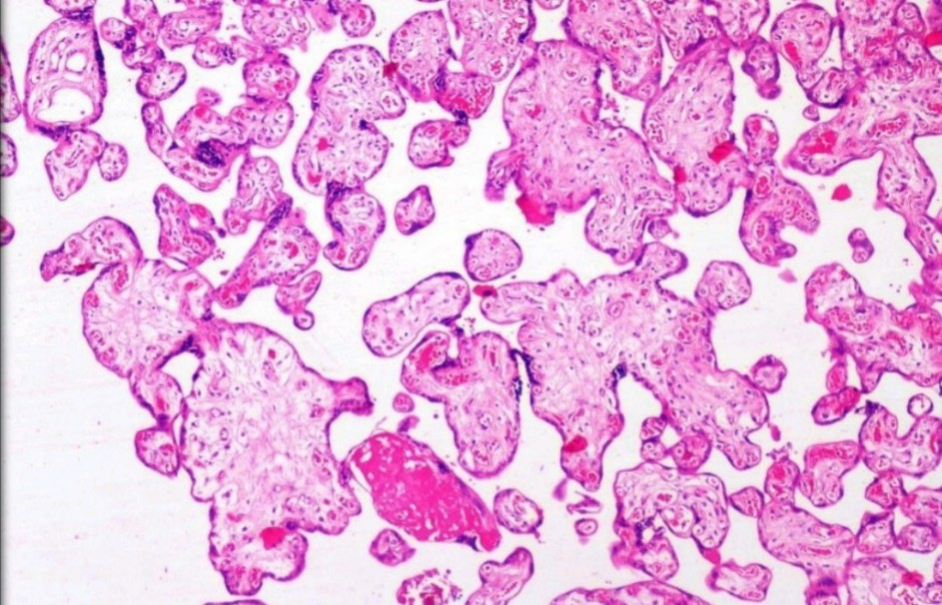
Distal villous immaturity – large immature villi with irregular outlines and loose reticular stroma. (H&E, 10 X)

This category included villous capillary lesion (9 cases) (Figure 10), villitis of unknown etiology (VUE-8 cases) (Figure 11), distal villous immaturity (DVI- 6 cases) (Figure 12), massive perivillous fibrin deposition (MPVFD- 4 cases), acute chorioamnionitis (3 cases) and abruption placenta (2 cases).

The clinical features of these miscellaneous causes are described in Table 7. 

**Table 7:** Clinical features associated with miscellaneous microscopic findings

Lesions		Villous capillary lesion (n=9)	VUE(n=8)	DVI(n=6)	MPVFD(n=4)	Acute ChorioAmnionitis(n=3)
Gestationalage	<37weeks	8	6	-	8	1
	>37weeks	1	2	6	-	2
Oligo hydroamnios		3	3	4	1	2
PIH		2	1	-	1	-
Premature Rupture Of Membrane		3	2	1	1	-
GDM		1	-	-	-	-
Uterine abnormality		-	1	-	-	-
Meconium stained liquor		-	1	-	-	1
Cardiac causes		-	-	1	1	-

## Discussion

Fetal growth restriction is one of the causes of perinatal morbidity and mortality. Placental insufficiency is the most common cause of FGR. Although there is a great advancement in technologies, diagnosing FGR is difficult and it can have many implications in the current as well as subsequent pregnancies. Hence, understanding the underlying placental pathology becomes important.

In this study, we analyzed and documented the clinicopathological features of 50 placentas of growth-restricted fetuses.

**Clinical Correlations**

More than 50% of the cases were primigravida and 65% were delivered as preterm, which is similar to a study by Mardi K. (13). Placentas of 46 cases were small for gestational age (SGA) and none were large for their gestational age. 

Many maternal co-morbidities were found to be associated with FGR, out of which oligohydramnios and PIH occupied the common causes. This correlated with studies conducted by So-Young Park* et al.* (14) and UjwalaCh* et al.* (15).

The placental coefficient is the ratio of placental weight to fetal weight. According to Little W, the normal placental coefficient ranges between 0.10 and 0.18 with a mean value of 0.14 (16). In our study, the placental co-efficient ranged between 0.06 and 0.27 with a mean of 0.14. Although the majority of placentas and fetuses were small for gestational age, the mean placental co-efficient value was normal. This explains that the placentas in a majority of the cases would have undergone adaptation to compensate for the placental insufficiency. A study by Fox H (17) also showed similar results.

**Umbilical Cord Abnormalities (n = 26)**

Hypercoiling of the cord followed by velamentous insertion were the two predominant umbilical cord abnormalities in our study population (Figures 1 & 2). Our study supports the hypothesis that any gross abnormality of the cord can cause mechanical compression resulting in reduced blood flow to the fetal vessels resulting in fetal growth restriction. A few cases showed hypo-coiling of the umbilical cord. Abnormal neurological development in growth-restricted fetuses may result in reduced Fetal movement, thereby producing hypo-coiling of the cord (18).

**Maternal Vascular Malperfusion (MVM) (n = 32)**

Maternal vascular malperfusion is the most common histopathological cause of FGR obtained in our study population. Redline* et al.* (19) showed that the sensitive predictors of placental hypoxia were placental site trophoblastic giant cells and cell islands while the more specific predictors were atherosis and muscularized arteries of the basal plate. In agreement, increased syncytial knots increased perivillous fibrin deposition and distal villous hypoplasia were the common pathological findings among our cases (Figures 3, 4, & 5).

The more specific predictors such as decidual vasculopathy included acute atherosis or fibrinoid necrosis of the vessel wall, villous agglutination, and placental site trophoblastic giant cells were seen in one-third of cases (Figure 6a &b). Since most MVM cases were preterm (<37 weeks) and showed features of distal villous hypoplasia, increased syncytial knots, perivillous fibrin deposition, and accelerated maturation, this indicated that the placental hypoxic changes would have started very early in pregnancy. These results suggest that the placental malperfusion associated with chronic ischemia and adaptive changes were significantly increased in preterm deliveries, thus explaining the occurrence of placental impairment in early-onset diseases.

**Fetal Vascular Malperfusion (FVM) (n = 10)**

FVM is the second most common histopathological cause of fetal growth restriction. Thrombosis of the fetal vessels reduces the blood supply leading to ischemia in the downstream villi resulting in fetal growth restriction or stillbirth depending on the severity of the lesion (Figures 7, 8a & 8b).

According to a study conducted by Salamudin* et al.* (20), placentas with FVM produced fetal growth restriction in twice the number of cases compared to that of normal placentas. In the present study, all the histopathological findings such as vascular ectasia, groups of avascular villi, villous stromal karyorrhexis, nucleated RBCs, and thrombi in fetal vessels reflect the fetal blood flow obstruction and the resultant placental hypoxia (Figure 9a & b).

As there was no specific gross lesion in cases of FVM in our study, submission of more sections from the placental parenchyma in suspected cases might be helpful to identify this heterogenous lesion. We found more completely avascular hyalinized villi that reflect the chronic occurrence of vascular obstruction over a prolonged period. More than two-thirds of the lesions were focal than regional, explaining that less severe obstruction resulted in FGR and not intrauterine Fetal demise.

Studies conducted by Kraus* et al.* (21) and Redline* et al.* (22) documented that severe cases of FVM were associated with neurodevelopmental abnormalities such as cerebral palsy, neonatal encephalopathy, and other thromboembolic diseases producing cerebral infarction. Therefore, a long-term follow-up of these children is necessary.

**Combined Lesions**

The concomitant lesions of FVM with MVM and FVM with umbilical cord abnormalities were noted. FVM produces placental hypoxia and ischemic changes in a slowly progressive pattern. But it brings forth the cumulative effects when it occurs simultaneously with other lesions such as MVM and umbilical cord abnormalities resulting in severe Fetal growth restriction or intrauterine Fetal demise (23).

**Villous Capillary Lesions (n = 9)**

This is a spectrum of overlapping lesions that include chorangiosis, chorangiomatosis, and chorangioma. In our study, these cases were associated with hypoxic states such as PIH, GDM, and maternal cardiac diseases and were seen particularly in preterm placentas. These associations were similar to a study conducted by Christina Bagby and Raymond W. Redline (24). These results suggest that placental vascular lesions may represent an abnormal response to hypoxia that has a high association with prematurity and FGR.

**Villitis of Unknown Etiology (VUE) (n = 8)**

16% of our FGR cases revealed VUE. The two hypotheses regarding the etiology of VUE are unidentified infection and graft versus host reaction. 

According to a study by Knox* et al.* (25) VUE was seen to be associated with recurrent abortions, FGR, intrauterine Fetal demise, and neurological impairment. Redline* et al.* (26) showed that VUE was also associated with recurrent villitis in subsequent pregnancies. Another study by Russell* et al.* (27) elucidated that the severity of FGR correlated with the severity (grade) of villitis. Recurrence was more often seen in high-grade multifocal/diffuse villitis. All of our VUE cases were of low grade except one that showed high-grade villitis with patchy distribution.

There were 2 cases, where the placenta showed combined lesions of obliterative vasculopathy and chronic villitis. This combination might have a cumulative effect either due to coagulopathy associated with VUE or immune-mediated fetal injury. Recognizing VUE in the index pregnancy may alert the clinician to prevent the complications and to treat with aspirin/ steroids in the next pregnancy.

**Distal Villous Immaturity (DVI) (n = 6)**

In our study, DVI cases were associated with oligohydramnios and maternal cardiac disease suggesting reduced uteroplacental circulation resulting in delayed villous maturation. In contrast to earlier studies, in this study, we found all the placentas with DVI were small for gestational ageDVI is a clinically challenging phenotype with recurrent risk in subsequent pregnancies. The outcomes associated with DVI are intrauterine Fetal demise, FGR, and chronic diseases in later life. Since there is no specific marker or imaging study to diagnose this entity antenatally, identifying this condition by analyzing the placentas may help prevent the catastrophic event in the subsequent pregnancy by implementing early induction of labor.

**Miscellaneous Causes**

***Massive Perivillous Fibrin Deposition (MPVFD)*** **(n = 4)**

MPVFD was one of the rare causes of FGR (8%), frequently presented with preterm delivery, and can sometimes produce recurrence in subsequent pregnancies (28). Women diagnosed with placental MPVFD with or without underlying autoimmunity should be treated with anticoagulants to improve the outcome of subsequent pregnancies.

***Acute Chorioamnionitis*** **(n = 3)**

Of FGR cases, 6% had acute chorioamnionitis. It can occur due to an infectious agent or due to immune-mediated pathology following exposure to meconium or fetal hypoxia as seen in FGR. The severity of maternal and fetal inflammatory responses correlates positively with the clinical significance. So, on diagnosing histologic acute chorioamnionitis with higher stage and grade inflammatory responses, the clinicians must be alerted immediately to aid in prompt treatment of the neonates. The clinicians may also utilize the frozen section study in cases that are clinically suspicious of acute chorioamnionitis

Although many causes produce fetal growth restriction individually, the severity depends on the cumulative effects of all the lesions. Hence, a meticulous examination should be done on all placentas submitted for histopathological analysis. Proper correlation with clinical details is also essential for a better understanding of the underlying pathology

## Limitations of This Study

A comparative study using gestational age-matched control placentas was not done. Future research in this field with a larger sample size may provide further insights into the aetiopathogenesis of FGR.

## Conclusion

Despite understanding the clinical significance of placental examination, the number of placentas sent for histopathological examination remains low. The increased incidence of multiple gestations, prematurity, intrauterine fetal demise, new reproductive technologies, and medico-legal disputes make placental examinations mandatory. The mystery of fetal malady can be explained by examining the placenta which may provide some answers to the clinicians and patients. Identification of recurrent placental lesions may also aid in the management of the current and subsequent pregnancies. This understanding of placental pathology raises the hope to control and prevent adverse perinatal outcomes (FGR, IUD) and treat the fetus in-utero in the future.

## Funding

This research received no specific grant from any funding agency in the public, commercial, or not-for-profit sectors.

## Conflict of Interest

There are no conflicting interests.
